# Depressive symptoms and early retirement intentions among Danish eldercare workers: Cross-sectional and longitudinal analyses

**DOI:** 10.1186/s12889-015-1973-1

**Published:** 2015-07-17

**Authors:** Mette Andersen Nexo, Vilhelm Borg, Camilla Sandal Sejbaek, Isabella Gomes Carneiro, Pernille U. Hjarsbech, Reiner Rugulies

**Affiliations:** The National Research Centre for the Working Environment, Lersoe Parkallé 105, DK-2100 Copenhagen, Denmark; Department of Public Health, University of Copenhagen, Oester Farimagsgade 5, Post box 2099, DK-1014 Copenhagen K, Denmark; Cancer Research UK partnership with the National Cancer Intelligence Network, Wellington House, 133-55 Waterloo Road, London, SE1 8UG U. K; Danish Institute for Local and Regional Government Research. Koebmagergade 22, DK-1150 Copenhagen K, Denmark; Department of Psychology, Copenhagen University, Oester Farigmagsgade 2A, DK-1353 Copenhagen, Denmark

## Abstract

**Background:**

Depression increases the risk of disability pension and represents a health related strain that pushes people out of the labour market. Although early voluntary retirement is an important alternative to disability pension, few studies have examined whether depressive symptoms incur early voluntary retirement. This study examined whether depressive symptoms and changes in depressive symptoms over time were associated with early retirement intentions.

**Methods:**

We used a cross-sectional (n = 4041) and a prospective (n = 2444) population from a longitudinal study on employees of the Danish eldercare sector. Depressive symptoms were measured by the Major Depression Inventory and the impact of different levels of depressive symptoms (severe, moderately severe, moderate, mild and none) and changes in depressive symptoms (worsened, improved, unaffected) on early retirement intentions were analysed with multinomial logistic regression.

**Results:**

In the cross-sectional analysis all levels of depressive symptoms were significantly associated with retirement intentions before the age of 62 years. Similar associations were found prospectively. Depressive symptoms and worsened depressive symptoms in the two year period from baseline to follow-up were also significantly associated with early retirement intentions before age 62. The prospective associations lost statistical significance when controlling for early retirement intentions at baseline.

**Conclusions:**

The whole spectrum of depressive symptoms represents a health related strain that can incur intentions to retire early by early voluntary retirement. In order to change the intentions to retire early, the work related consequences of depressive symptoms should be addressed as early in the treatment process as possible.

## Background

The ageing work force challenges the economy of most Western countries and calls for new strategies to maintain people in the work force. Depression is a common mental disorder [1, 2] and is one of the leading causes of early retirement by disability pension in many Western Countries [2–12]. Although it is more common to retire early by other forms of retirement schemes than disability pension, it is poorly understood how depressive symptoms affect early voluntary retirement (EVR) plans.

EVR can be defined as early retirement by either self-financed or publicly financed non-illness based pensions. Publicly financed EVR is available in a number of Western countries and is generally available at the age of 60 years or earlier [13]. Publicly financed EVR makes it economically viable for people to retire early. In 2001 in Denmark, approximately 40 % of all citizens aged 60–64 years retired early by EVR [14]. Recently, most Western countries have gradually raised the official retirement age and the age at which publicly financed EVR is available [15]. Despite such restrictions, EVR remains the most common way of retiring early in Denmark [16–18].

Both ‘pull’ and ‘push’ factors can help explain people’s incentives to retire early [19]. Factors that ‘pull’ people out of the labour market are mostly related to the prospect of a pleasurable retirement, such as a wish for self-actualization later in life or more time for leisure activities [20] *e.g.*, being female and having a spouse that is retired have been associated with EVR [21]. Factors that ‘push’ people out of the labour market are often driven by a need to retire to relieve strain from, *e.g.*, experienced health and medical problems [22, 23], increasing age, a poor work environment [24] or unemployment [17, 20]. In this regard, depression can be considered a push factor.

Depression is mostly characterised by an episodic course, but there is a high risk of relapse [25]. The core symptoms of depression are persistent low mood and marked loss of pleasure in usual activities [26, 27]. The symptoms affect most aspects of functioning including the ability to perform at work [7, 28, 29] and often incur absence from work [30, 31]. Some symptoms and work disability can persist after a clinical depression has been treated [7, 28]. Two recent prospective studies found that having a depression increased the risk of EVR years later [32, 33] while one of the studies [32] found the same, but a less strong effect for sub-threshold depression, *i.e.*, people experiencing symptoms not severe enough to reach the threshold of a clinical depression.

These prospective studies were based on between-group differences, *i.e.*, people with depression were more likely to retire early than people without depression. By contrast, a study of within-person changes would measure whether changes in depression in the same person from one point in time to another affected early retirement [34]. Within-person measurements are needed in order to clarify whether improvements in depressive symptoms can change the intention to retire early.

Early retirement plans are likely to change over the course of one’s life and can be viewed as a transitional process. A process which often begins with considerations and early retirement intentions then proceeds to a decision to retire early and eventually ends with actual retirement [35, 36]. Most of the longitudinal studies have examined the association between mental health and actual EVR [22, 23, 37, 38]. In order to prevent EVR due to mental health problems, it is important to identify factors that influence early retirement as early in the decision making process as possible. Two recent cross-sectional studies showed that poor mental health was strongly associated with early retirement intentions [37, 38]. However, in order to clarify whether the retirement intentions were maintained over time, this association should be examined prospectively.

This study examined the association between depressive symptoms and early retirement intentions in four research questions:

1. Is the severity of depressive symptoms associated with immediate early retirement intentions?
2. Is the severity of depressive symptoms associated with early retirement intentions over a two-year period?
3. Are changes in depressive symptoms associated with early retirement intentions over a two-year period?
4. Do depressive symptoms affect changes in retirement intentions over time?

## Methods

### Population

This study used data from a prospective study of eldercare workers health and work characteristics [39]. Questionnaires were sent to all employees in the eldercare sector of 36 municipalities in Denmark over three waves: 2004/05 (Baseline), 2006/07 (Time 1: T1), and 2008/09 (Time 2: T2). Response rate at baseline was 78 % accounting for 9949 of 12746 possible employees that answered the questionnaire. At T1, 10029 out of the 15697 possible employees responded (response rate = 64 %) and at T2, 8431 of 13945 possible employees responded (response rate = 61 %). Further details of this study can be found elsewhere [39, 40]. In this study, we only used data from the 2006/07 and 2008/09 waves. The mean follow-up time was 24 months ranging between 22–26 months. We selected two study populations for the present study: A cross-sectional and a longitudinal population.

In the cross-sectional study population, we included participants who responded at T2 (n = 8431). We excluded those participants who were below 45 or above 59 years at T2 (n = 3490) and those who had missing values (n = 145), or who answered “I don’t know” to the question about early retirement intentions (n = 755). The final cross-sectional population amounted to 4041. We did not have information on actual retirement, but in order to account for the possible bias of people who left the population because of EVR the participants were excluded at age 60 at T2. In Denmark, most employees can receive publicly financed EVR at the age of 60 (Danish: Efterløn) and the publicly financed old age pension at age 65 (Danish: Folkepension). Due to high Danish taxes on income self-financed retirement before the age of 60 is rare.

In the prospective study population, we included those participants who responded at both T1 and T2 (n = 5206). We excluded participants who were below 45 or above 57 years old at T1 (*i.e.*, above 59 years at T2, n = 2336) and who had turned 60 years at T2 (n = 11). We also excluded participants, who had missing values (n = 25), or answered “I don’t know” (n = 390) to the question about early retirement intentions. The final prospective population consisted of 2444 employees.

This study was approved by the Danish Data Protection Agency. In Denmark, questionnaire studies require approval by the Danish Data Protection Agency, but not by an ethical committee. International ethical guidelines were nevertheless followed. Participants were informed about the study prior to participation and they were informed that participation was voluntary and that they could withdraw from the study at any time. They were also assured confidentiality.

### Depressive symptoms

We measured depressive symptoms by the Major Depression Inventory (MDI). The MDI is a widely used self-rating scale to asses depression [41] according to both the DSM-IV [26] and ICD-10 [27] diagnostic classifications. The MDI has been validated in a Danish clinical setting and in the general population in Denmark [42, 43]. The MDI consists of 10 items that assess if depressive symptoms are present, for instance: “*Have you felt low in spirits or sad”?* or “*Have you lost interest in your daily activities?”.* Using a six-point Likert scale, the items measure how frequent the symptoms have been present during the past two weeks (0 = The symptom is not present at all; 5 = The symptom is present all the time). The individual responses to the items are summed up to a severity scale that ranges from 0 to 50 - the higher the score the more severe the depressive symptoms. A cut-off score of ≥20 indicate a probable major depression. We categorised the MDI scores at T1 into five different categories indicating the severity of depressive symptoms:

1. ‘Severe depressive symptoms’ (scores ≥20);
2. ‘Moderately severe depressive symptoms’ (scores between 15 and 19);
3. ‘Moderate depressive symptoms’ (scores between 10 and 14);
4. ‘Mild depressive symptoms’ (scores between 5 and 9);
5. ‘No depressive symptoms’ (scores between 0 and 4).

Although a self-report questionnaire is able to indicate the presence of a clinical depression, a valid diagnosis can only be made by clinical assessment performed by a health care professional [26]. We therefore chose to describe the categories with a terminology referring to severity of depressive symptoms as opposed to categories such as ‘major depression’ or ‘sub-threshold‘depression. Severity of depressive symptoms at T2 was used as a predictor variable in the cross-sectional analyses.

Severity of depressive symptoms at T1 and changes in depressive symptoms from T1 to T2 were used as predictor variables in the longitudinal analyses. Changes in depressive symptoms had three categories: “Worsened”, “improved” or “unaffected” symptoms. Participants were classified with “worsened symptoms” if their MDI score had increased five points or more and with “improved symptoms” if their MDI-score had decreased by five points or more. All other participants were classified with “unaffected symptoms”.

### Outcome

The intention to retire early was measured with a single item: *“When would you like to retire from the labour market?* Five response categories indicated age of intended retirement: “*I would like to work until I turn 65 years old”, “I would like to receive early retirement pension, when I am between 62 and 65 years old”*, *“I would like to receive early retirement pension, when I am between 60 and 62 years old”,* “*I would like to retire, before I turn 60 years old*” or, “*I don’t know*” These were recoded into three categories:

1. Very early retirement intentions, which included the responses: ‘*I would like to retire before the age of 60’* and *‘when I am between 60 and 62 years old’*;

2. Early retirement intentions, which included the response: ‘*I would like to retire when I am between 62 and 64 years old’;*

3. Normal retirement intentions, which included the response: ‘*I would like to work until I turn 65 years old’;*

Participants who answered “*I don’t know”* were excluded from the study populations, as described previously.

### Statistical analyses

Statistical analysis was conducted in PASW 18 (formally SPSS). Using multinomial logistic regression for the cross-sectional and prospective analyses, we examined the association of depressive symptoms with the probability of very early and early retirement intentions. A p-value of <0.05 was regarded as statistically significant in these analyses. The cross-sectional analysis was adjusted for gender, age, marital status, working hours, seniority and type of occupation. We tested two models in the prospective analyses. Model 1 was adjusted for the same variables as the cross-sectional analysis. Model 2 was further adjusted for early retirement intentions at T1. The drop-out analyses were conducted using Pearson chi-square (*χ*^2^).

## Results

### Characteristics of study populations

Table 1 shows the characteristics of the study population from the cross-sectional and longitudinal analyses.

**Table 1:** Characteristics of study populations (% = percentage)

Populations						
	Longitudinal				Cross-sectional	
	n = 2444				n = 4041	
	n at T1	%	n at T2	%	n at T2	%
Depressive symptoms	MD = 153	MD = 6	MD = 171	MD = 7	MD = 305	MD = 8
Severe (MDI > =20)	123	5	128	5	220	5
Moderately severe (MDI 15–19)	139	6	139	6	234	6
Moderate (MDI 10–14)	323	13	350	14	564	14
Mild (MDI 5–9)	841	34	794	33	1293	32
None (MDI 0–4)	865	35	862	35	1425	35
Change in depressive symptoms			MD = 296	MD = 12		
Worsened			360	15		
Improved			331	13		
Unaffected			1457	60		
Retirement intentions	MD = 21	MD = 8				
Very early (before 62)	1357	56	1370	56	2136	53
Early (62 and 64 years)	580	24	726	30	1216	30
Normal retirement (65 years)	286	12	348	14	689	17
Age						
45-49 years	845	35			1116	28
50-54 years	1024	42			1413	35
55-60 year	575	24			1512	37
Gender						
Male	97	4			220	5
Female	2347	96			3821	95
Marital status	MD = 81	MD = 3			MD = 61	MD = 1
Cohabitant	1966	81			3219	80
No cohabitant	397	16			761	19
Shift work	MD = 30	MD = 1			MD = 28	MD = 1
Day shift	1595	65			2620	65
Night shift	819	34			1393	34
Work experience	MD = 39	MD = 2			MD = 407	MD = 10
0-5 years	865	35			1549	38
6-15 years	874	36			1069	27
> = 16 years	666	27			1016	25
Type of occupation	MD = 15	MD = 1			MD = 25	MD = 1
Supervisor	241	10			405	10
Nurse or therapist	258	10			457	11
Care helpers and assistants	1702	70			2745	68
Other	225	9			409	10

MD = Missing data

### Cross-sectional analysis

All levels of the elevated depressive symptoms were significantly associated with very early retirement intentions before age 62 (Table 2). Only mild and moderate levels of depressive symptoms, but not moderately severe or severe depressive symptoms, were significantly associated with early retirement intentions between ages 62 to 64.

**Table 2:** Cross-sectional analysis. Adjusted odds ratios (OR) for depressive symptoms at T2 and early retirement intentions at T2 (n = 4041)

	Early retirement intentions			
	Very early		Early	
	(before age 62)		(age 62 to 64)	
Depressive symptom at time 2	OR	(95 % Cl)	OR	(95 % Cl)
Severe (MDI > =20)	**2.11**	**(1.36-3.26)**	0.90	(0.54-1.50)
Moderate severe (MDI 15–19)	**2.07**	**(1.34-3.19)**	1.16	(0.72-1.88)
Moderate (MDI 10–14)	**2.03**	**(1.48-2.80)**	**1.58**	**(1.13-2.21)**
Mild (MDI 5–9)	**1.59**	**(1.26-2.01)**	**1.44**	**(1.13-1.84)**
None (MDI 0–4)	1		1	

**Significant in bold**

MDI: Major Depression Index

*Reference group: Retirement at 65 years or above

OR adjusted for: Gender, age, marital status, shift work, work experience, type of occupation

### Prospective analysis

‘Moderately severe’, ‘moderate’, and ‘mild’- but not a ‘severe’ level of depressive symptoms at T1 were significantly associated with very early retirement intentions a T2 (Table 3). Only a ‘mild’ level of depressive symptoms was significantly associated with early retirement intentions between age 62 and 64.

**Table 3:** Prospective analyses. Adjusted odds ratios (OR) for depressive symptoms at T1, changes in depressive symptoms from T1 to T2, and early retirement intentions at T2 (n = 2444)

	Early retirement intentions (T2)							
	Model 1#				Model 2##			
	Very early		Early		Very early		Early	
	(before age 62)*		(age 62 to 64)*		(before age 62)*		age 62 to 64)*	
	OR	(95 % Cl)	OR	(95 % Cl)	OR	(95 % Cl)	OR	(95 % Cl)
Depressive symptoms at T1								
Severe (MDI > =20)	1.87	(0.87-4.03)	1.31	(0.57-2.97)	1.99	(0.57-7.00)	1.64	(0.52-5.21)
Moderate severe (15–19)	**2.05**	(1.02-4.09)	0.93	(0.42-2.05)	1.76	(0.59-5.28)	0.84	(0.29-2.39)
(MDI 10–14)	**1.65**	(1.02-2.66)	1.46	(0.87-2.44)	0.77	(0.38-1.56)	0.89	(0.46-1.72)
Mild (MDI 5–9)	**1.51**	**(1.08-2.07)**	**1.72**	**(1.22-2.41)**	0.98	(0.60-1.59)	1.21	(0.78-1.88)
None (MDI 0–4)	1		1		1		1	
Change in depressive symptoms (from T1 to T2)								
Worsened	**1.93**	**(1.27-2.94)**	1.49	(0.96-2.33)	1.32	(0.72-2.43)	1.18	(0.67-2.09)
Improved	1.34	(0.82-2.22)	1.33	(0.78-2.25)	1.31	(0.63-2.70)	1.31	(0.66-2.55)
Unchanged	1		1		1		1	

**Significant in bold**

MDI: Major Depression Index

*Reference group: Retirement at 65 years or above

# OR adjusted for: Gender, age, marital status, shift work, work experience, type of occupation

## OR adjusted fro: Gender, age, marital status, shift work, work experience, type of occupation and early retirement intentions at T1

Worsened depressive symptoms from T1 to T2 significantly increased the risk of very early retirement intentions at T2, but not early retirement intentions (Table 3). Although not significant, there was a tendency that participants, who had improved their depressive symptoms, also had increased risk of early retirement intentions, compared to those who had unaffected symptoms.

After adjusting for retirement intentions at T1 (model 2, Table 3) all of the prospective associations found in model 1 were no longer statistically significant.

### Attrition analyses

The distribution of depressive symptoms in the selected cross-sectional and longitudinal study populations (Table 1) was similar to the entire population of responders at T1 and T2. The percentage (%) of all responders who had “severe” depressive symptoms at T1 (n = 10029) was 6 % and the percentage of all responders at T2 (n = 8431) was also 6 %. The percentage of all the responders represented in the other categories of depressive symptoms: “Moderately severe” T1 = 6 % and T2 = 6 %; “moderate” T1 = 13 % and T2 = 14 %; “mild” at T1 = 32 % and T2 = 31 %; “none” at T1 = 36 % and at T2 = 34 %; and missing data of all responders at T1 = 7 % and at T2 = 9 %.

The sample of dropouts consisted of those participants between 45–57 years at T1, who had not turned 60 at T2 and who had returned the questionnaire at T1 but not at T2 (n = 1467). Of this sample, 37 % (n = 541) did not return the questionnaire because they no longer worked at the particular workplace. Chi-square tests revealed that these non-responders were more likely to be married (p < 0.05), be male (p < 0.001), and have less years of work experience (p < 0.001) compared to the responders from the prospective study population. No differences between depressive symptoms, age, shift work, or type of occupation were found.

Chi-square tests revealed that the non-responders who remained at the same work place (n = 926) were more likely to be male (p < 0.001), to be younger (p < 0.05), have less years of work experience (p < 0.05), and to have more severe depressive symptoms (p < 0.001) compared to the responders from the selected prospective study population. No differences between marital status, shift work, or type of occupation were found.

Chi-square tests revealed that the participants who were excluded because they had answered “I don’t know” to the question about early retirement intentions from both the cross-sectional and longitudinal study populations were significantly younger (p < 0.05) than the study participants, but did not significantly differ with regards to depressive symptoms, gender, marital status, shift work, or work experience.

## Discussion

The results from the cross-sectional analysis showed that all levels of elevated depressive symptoms doubled the chance of very early retirement intentions (before age 62), but not early retirement intentions (between ages 62 and 64). The same association was also found when examined over time, except that ‘severe depressive symptoms’ did not significantly affect intentions to retire before age 62. In addition, those who experienced worsened depressive symptoms over time were also more likely to have very early retirement intentions compared to those with no change in depressive symptoms. However, when adjusting for early retirement intentions at T1 in the longitudinal analyses, the results were no longer significant.

Our findings are in line with previous studies showing that depressive symptoms increase the risk of early retirement by either EVR [32, 33, 37, 38] or disability pension [8–11]. Whereas previous studies examined this association between groups only, we were also able to examine whether changes in depressive symptoms in the same person from one point in time to another influenced early retirement intentions. Worsened depressive symptoms were prospectively associated with very early retirement intentions [Odds Ratio: 1.93, 95 % CI 1.27-2.94]. We also found some indication that the early retirement intentions remained even after the depressive symptoms were relieved [Odds Ratio: 1.34, 95 % CI 0.82-2.22], but these findings were not significant and therefore remains speculative.

We were also able to examine the association between depressive symptoms and changes in retirement intentions from T1 to T2. The prospective associations were no longer statistically significant when adjusting for retirement intentions at T1, regardless of the severity of depressive symptoms at T1 or whether the depressive symptoms had worsened or improved. A significant result after adjusting for retirement intentions at T1 would require that the retirement intentions increased from T1 to T2 among those participants who already had depressive symptoms and early retirement intentions at T1. Our finding indicated that the association between depressive symptoms and retirement intentions remained the same or was weakened in the two-year period.

In accordance with other recent studies, our study indicated that the presence of even mild depressive symptoms had significant work related consequences [30, 32, 44], including decisions to retire early by EVR [32]. The prospective, but not the cross-sectional, analyses failed to show that a severe level of depressive symptoms (indicating probable major depression) predicted early retirement, which contradicted the results of two previous studies [32, 33]. In our study, more non-responders were represented in the severe depressive symptoms category, which might have weakened the associations and might explain the non-significant findings.

It remains unknown whether the impact of depressive symptoms that are below the threshold of a clinical depression on early retirement should be understood as the effect of residual symptoms from a previous depression or as symptoms that reoccur independently. Our results in either case show that it is important to consider the whole spectrum of depression when analysing the impact of depression on EVR over time.

Our results may have been confounded by ‘push’ and ‘pull’ factors at T1. Studies have identified several factors that play an important role in both early retirement intentions and actual early retirement, such as an adverse working environment [24, 37, 38], spouses retirement status [45], being female [21], older age [46], and poor self-rated health and medical problems [22, 23]. We adjusted the results for most of these factors, but lack of control for comorbidity was an important limitation in our study. Two recent studies showed that the association of mental health and early retirement intentions remained even after adjusting for physical health, socioeconomic status, and spouses employment status [37, 38]. Comorbidity is, nevertheless, an important confounder that, had we controlled for it, could have weakened the associations.

Another important limitation of this study was the selective attrition. The response rate from the original study was fairly high [47], but we had to further restrict the study sample to only include participants between ages 45 and 59. Although selection bias was not introduced by excluding people from the study sample because of age, we also excluded a large proportion of the participants from the longitudinal sample because they had responded at T1, but not at T2.

Our attrition analyses showed that 37 % of non-responders no longer worked at the particular work place. Early retirement before the age of 60 is rare in Denmark [16, 48, 49] and since the eldercare sector in Denmark is characterised by a high degree of job change [50], it is likely that these non-responders dropped out of the study because they had changed jobs. However, our attrition analyses also showed that the non-responders who remained at the same workplace were more likely to experience severe depressive symptoms than responders. This attrition can therefore have led to an underestimation of the findings.

The study population consisted of mainly female eldercare workers, an occupational group that is known for reporting a lower quality of the work environment compared to other sectors [39]. Thus, it is possible that our study population was more likely to have early retirement intentions than the general workforce. We attempted to account for this limitation by controlling for gender and work characteristics, but our results are primarily generalisable to older care workers in Western countries.

## Conclusions

The whole spectrum of depressive symptoms represents a health related strain that can influence EVR. Our findings suggest that the severity of depressive symptoms can have an immediate impact on the intentions to retire early and that these intentions are maintained, but weakened over time. These findings also suggest that in order to change the intentions to retire early, the work related consequences of depressive symptoms should be addressed as early in the treatment process as possible.
